# Imaging of haemodialysis: renal and extrarenal findings

**DOI:** 10.1007/s13244-015-0383-3

**Published:** 2015-02-14

**Authors:** Ferruccio Degrassi, Emilio Quaia, Paola Martingano, Marco Cavallaro, Maria Assunta Cova

**Affiliations:** Department of Radiology, Cattinara Hospital, University of Trieste, Strada di Fiume 447, 34149 Trieste, Italy

**Keywords:** Haemodialysis, Acquired cystic kidney disease, Renal neoplasm, Renal osteodystrophy, Dialysis access complications

## Abstract

Electrolyte alterations and extra-renal disorders are quite frequent in patients undergoing haemodialysis or peritoneal dialysis. The native kidneys may be the site of important pathologies in patients undergoing dialysis, especially in the form of acquired renal cystic disease with frequent malignant transformation. Renal neoplasms represents an important complication of haemodialysis-associated acquired cystic kidney disease and imaging surveillance is suggested. Extra-renal complications include renal osteodistrophy, brown tumours, and thoracic and cardiovascular complications. Other important fields in which imaging techniques may provide important informations are arteriovenous fistula and graft complications.

*Teaching points*

• *Renal neoplasms represent a dreaded complication of haemodialysis*.

• *In renal osteodystrophy bone resorption typically manifests along the middle phalanges*.

• *Brown tumours are well-defined lytic lesions radiographically*, *possibly causing bone expansion*.

• *Vascular calcifications are very common in patients undergoing haemodialysis*.

• *Principal complications of the AV fistula consist of thrombosis*, *aneurysms and pseudoaneurysms*.

## Introduction

End-stage renal disease occurs when chronic renal failure progresses to the point that the kidneys are permanently functioning at less than 10 % of their capacity, namely the glomerular filtration rate (GFR) is permanently lower than 15 ml/min/1.73 m^2^. End-stage renal disease used to be a lethal condition until the advent of long-term haemodialysis and renal transplantation. In the USA, diabetic nephropathy, hypertension and glomerulonephritis cause approximately 75 % of end-stage renal disease in patients undergoing haemodialysis.

The dialysis is the exchange of solutes and waste products via either an extracorporeal membrane (haemodialysis) or a peritoneal membrane (continuous ambulatory or cycling peritoneal dialysis). As far as haemodialysis is concerned, the solute removal primarily depends on passive diffusion across a semipermeable membrane. Blood and dialysate flow rates are adjusted to replenish the supply of incoming solute available for diffusion [1]. The efficiency of ultrafiltration depends on the water permeability of the device and the surface area [1]. Haemodialysis has a higher clearance rate for low-molecular-weight substances such as urea [1]. Peritoneal dialysis should be performed in infants and patients with relative contraindications to haemodialysis such as severe vascular disease, active bleeding, haemorrhagic diathesis or cardiovascular instability.

The clinical constellation of signs and symptoms of end-stage renal disease is known as uraemic syndrome. Various problems related to the vascular access in patients on haemodialysis [2] are also common since the peritoneal dialysis catheter leads to the risk of peritonitis and local infections. Patients with end-stage renal failure are susceptible to all the complications of any underlying condition such as diabetes and hypertension as well as other metabolic and physiologic derangements. In addition, chronic immunosuppression makes patients with end-stage renal disease prone to infections, tuberculosis included [3].

Imaging plays an essential role in the assessment of the possible renal and extra-renal complications in patients undergoing long-term haemodialysis.

## Chronic renal failure

Chronic renal failure can result from numerous renal parenchymal diseases, the most common being glomerulonephritis, diabetes, hypertension and the polycystic kidney disease. Diabetes and hypertension are now recognised as the leading causes of chronic renal failure in the USA [4]. Often, patients only seek medical attention when the renal disease has progressed to the uraemic stage since adult patients are usually unaware of their advancing renal failure until the GFR has decreased to less than 15 ml per minute, namely end-stage renal disease [4].

**Fig. 1:**
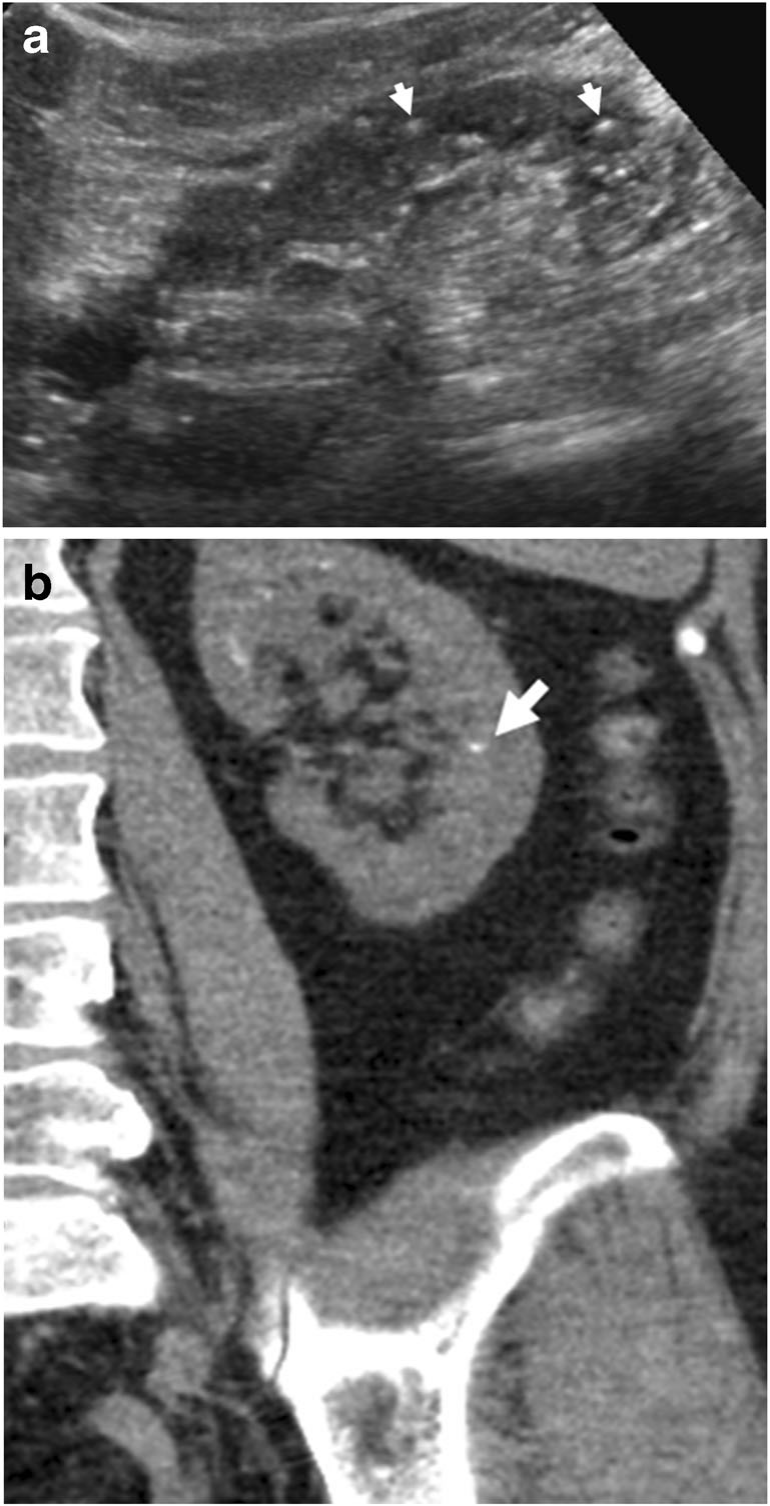
**a**–**b**: **a** Grey-scale US and (**b**) unenhanced CT, coronal reformations. Papillary calcifications in a patient undergoing long-term dialysis, appearing as hyperchoic spots on renal pyramids. Papillary calcifications may also be observed in analgesic nephropathy, in sarcoidosis, primary hyperparathyroidism, diabetes mellitus and medullary sponge kidney

**Fig. 2:**
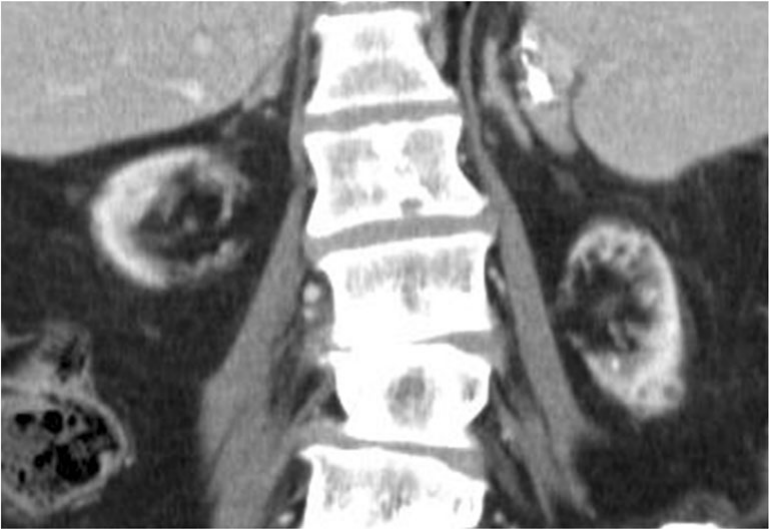
Chronic renal failure in a 76-year-old female. CT image of kidneys reveals a short length and diffuse reduction of renal cortical thickness

Ultrasound (US) with Doppler US examination of intrarenal vessels is the imaging modality of choice to be employed in patients with renal failure and is commonly performed early in the clinical course [5]. Chronic renal failure manifests with a reduction of renal dimensions that develops over months or years. US reveals a reduced renal length and reduced renal cortical thickness with an increased renal cortical echogenicity often associated with poor visibility of the renal pyramids and the renal sinus, marginal irregularities, parenchymal cysts and also papillary calcifications (Fig. 1a–b). Colour Doppler US reveals a reduced renal vascularity and increased RI values measured at the level of the segmental and interlobular arteries [6, 7] according to the stage of chronic renal failure. The capability of renal RI to aid in the prediction of renal dysfunction progression has been demonstrated. US follow-up of native scarred kidneys is indicated in patients with chronic renal failure treated with dialysis or renal transplantation because they develop the acquired cystic kidney disease (ACKD) with a significantly increased risk of solid and cystic renal malignancies. CT confirms the US findings by revealing reduced renal length and cortical thickness (Fig. 2).

## Renal abnormalities in long-term haemodialysis

### Acquired cystic kidney disease

**Fig. 3:**
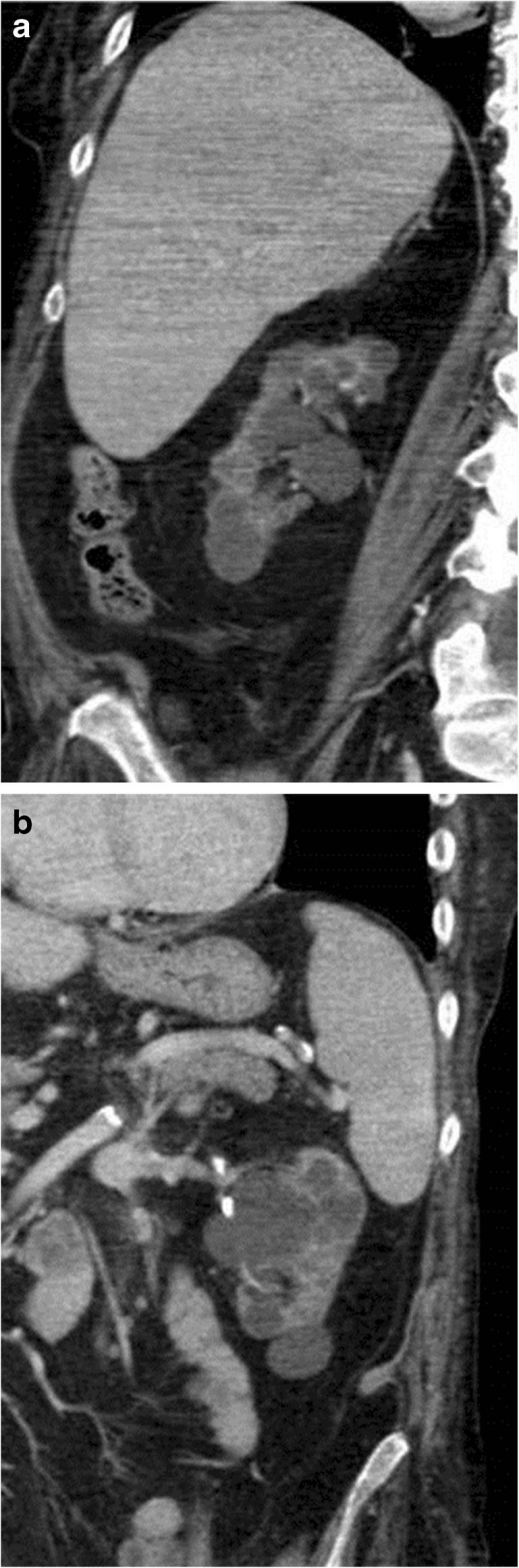
**a**, **b**: Unenhanced (**a**) and contrast-enhanced CT (**b**). Acquired cystic kidney disease in a patient undergoing long-term haemodialysis

**Fig. 4:**
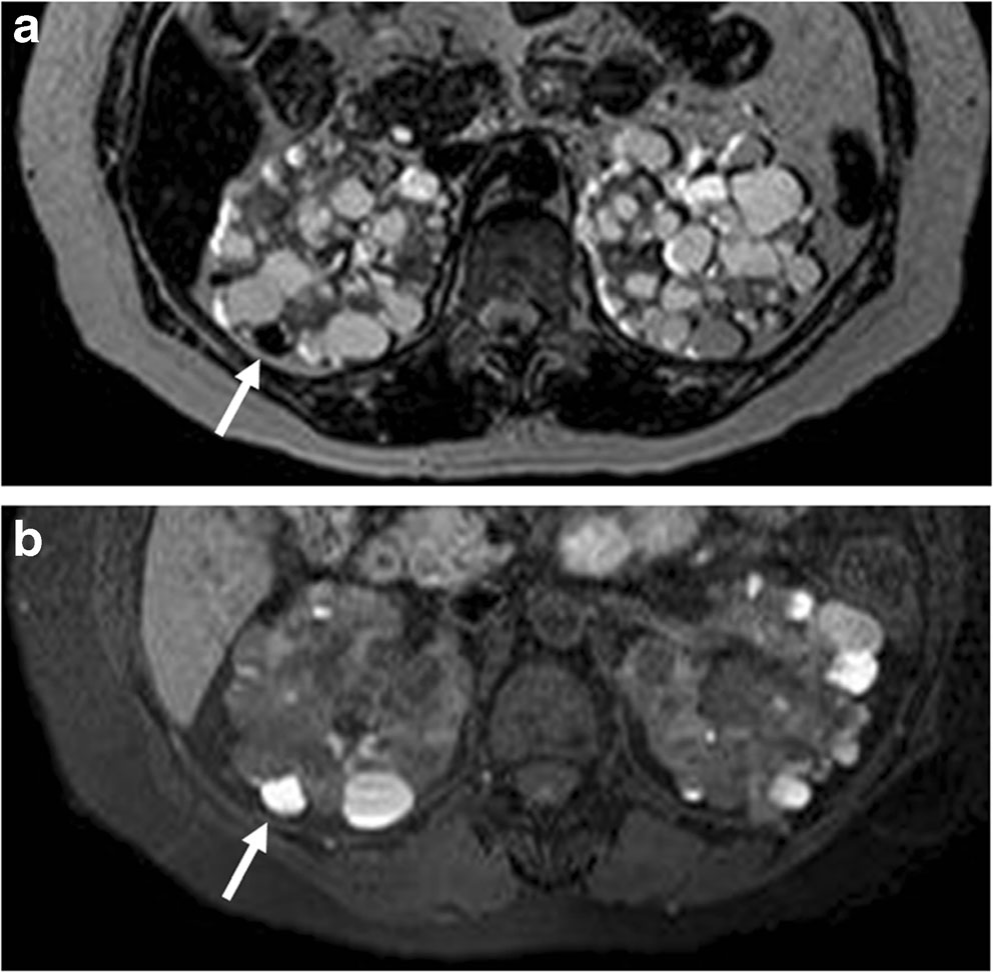
**a**, **b**: Haemorrhagic renal cyst (*arrow*) in the right kidney in a patient undergoing long-term haemodialysis. The cyst appears hypointense on T2-weighted MR sequences (**a**) and hyperintense on T1-weighted MR sequences (**b**)

ACKD was first described by Dunnil et al. (1977) and is characterised by the development of numerous fluid-filled cysts in patients with end-stage renal disease who have undergone prolonged dialysis but without a history of hereditary cystic disease (Fig. 3a, b). After 1–3 years of haemodialysis, 10–20 % of patients have ACKD, 40–60 % after 3–5 years of haemodyalisis and more than 90 % after 5–10 years of haemodialysis. The cysts measure 0.5 to 2 cm in diameter, contain clear fluid, are lined by either a hyperplastic or a flattened tubular epithelium, and often contain calcium oxalate crystals. They probably form as a result of obstruction of tubules by interstitial fibrosis or oxalate crystals. Frequently, some cysts may develop a haemorrhagic pattern causing haematuria (Fig. 4a, b). The likelihood of the development of ACKD increases with the duration of haemodialysis, and it is higher in male and older patients. Other factors such as the underlying renal insufficiency and serum creatinine level have no significant influence on the development of ACKD. The development of renal cell carcinoma in the wall of the cysts is the most serious complication of ACKD. The prevalence of renal cell carcinoma in patients with dialysis-associated ACKD undergoing long-term haemodialysis is increased, being in the range of 3–6 %. The prevalence is similar in patients with ACKD undergoing renal transplantation.

### Renal neoplasms

An increased incidence of renal tumours has been reported in patients with end-stage renal disease [8]. Renal cell carcinoma (RCC) presents with a frequency three to six times higher in patients receiving haemodialysis than in the general population [8]. Observations provide histological evidence that renal cell neoplasms are prone to develop in relatively young patients with renal failure when their uraemia is treated by long-term dialysis. The incidence of renal carcinoma increases with the duration of dialysis [8].

Other studies indicate that the stimulus for neoplastic growth comes with a cystic transformation of the kidneys. A very strong association with ACKD and an increased incidence of papillary renal tumours have been observed in many studies [9]. More than 80 % of all patients with end-stage renal disease developing a renal tumour actually have underlying ACKD [8]. Since these tumours are multiple and very often small in size, the ratio of histological RCC subtypes is shifted toward papillary renal carcinoma and papillary renal cell adenoma [8].

**Fig. 5:**
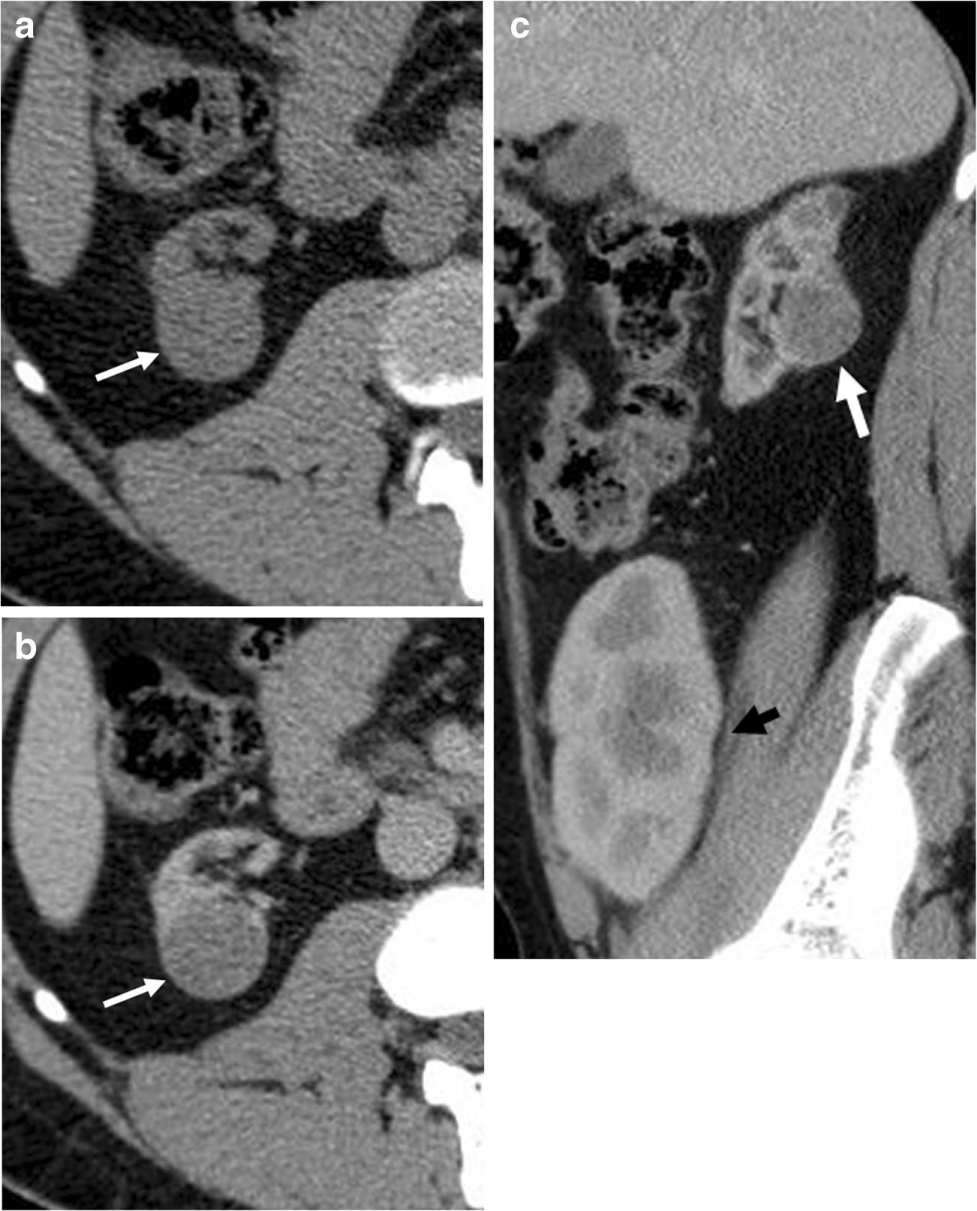
**a**–**c**: Unenhanced (**a**) and contrast-enhanced CT, transverse plane (**b**) and sagittal reformation (**c**). Rounded lesion (white arrow) of the right kidney in a patient after renal allograft transplantation (*black arrow*). The lesion shows indeterminate enhancement (an increase of 10 HU after contrast injection). The dissected specimen proved to be a papillary renal cell carcinoma. There was no sign of acquired cystic renal disease

Renal tumours developing in patients undergoing haemodialysis present at an earlier age compared to the general population, with a higher incidence in males, [8, 10–12], and they frequently are bilateral (9 %) and multi-centric (50 %) [5]. The papillary hyperplasia in the cyst is thought to be a precursor of the papillary carcinoma. Papillary-cell renal cell carcinoma accounts for about half the cases identified in patients undergoing haemodialysis, while it does not exceed 10 % in the general population (Fig. 5a–c) [13–16].

**Fig. 6:**
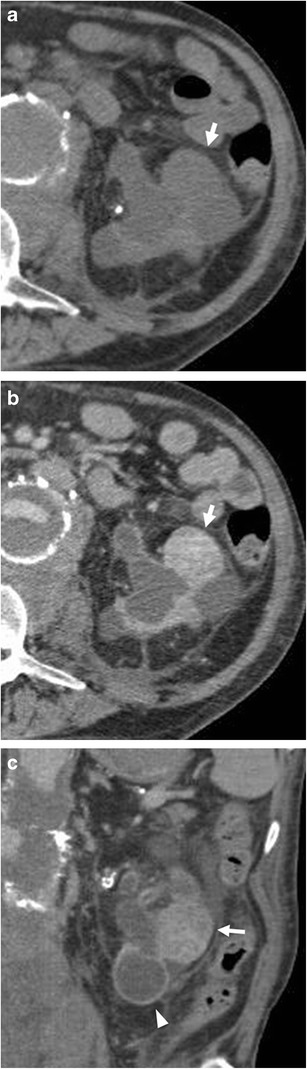
**a**–**c**: ACKD in a patient who has been undergoing haemodialysis for 10 years. **a** Unenhanced and contrast-enhanced CT, transverse plane (**b**) and coronal reformation (**c**). Both kidneys present multiple cysts; one of these at the inferior pole of the left kidney presents uniform thickening of the wall (Bosniak IIF) (*arrowhead*). Moreover a solid enhancing lesion, about 3 cm in size, is evident on the left kidney (*arrow*) corresponding to a clear-cell renal cell carcinoma

CT seems to provide better diagnostic accuracy than US or MR imaging in the detection of renal tumours in patients undergoing haemodialysis (Fig. 6a–c) [17, 18].

The leading sign of the tumour is the space occupied by the lesion localised in the atrophic renal parenchyma; the size of the distinguishable tumour is about 5 mm in diameter when causing the bulging of the renal surface [9]. Smaller lesions under the kidney surface may remain undetected, especially when ultrasound or plain CT is used [19]. Renal parenchymal atrophy in the end stage of glomerulonephritis or interstitial nephritis is the main limitation of ultrasound in imaging the kidneys in dialysis patients [9], while the enhancement of small nodular foci could help identify smaller tumours inside the renal parenchyma if multi-detector row computed tomography with isotropic resolution is used [9].

It is generally agreed that there is a need for regular surveillance of patients with ACKD for the early detection of renal cell carcinoma [17, 18].

Although regular follow-up trans-abdominal ultrasound or conventional helical CT is able to detect larger masses, Ferda et al. have shown that the capabilities of multi-detector row computed tomography offer more accurate imaging of multiple renal tumours in ESRD patients [9]. However, some authors maintain that the performance of periodic ultrasound controls allows achieving the early diagnosis and treatment of tumours of the kidney in patients in dialysis [20].

There is no clear consensus about screening in the long-term dialysis population. The current literature indicates that most patients will develop acquired renal cysts after 3–7 years on dialysis, the latter associated with a 1.6–7.0 % incidence of RCC. This has led some authors to recommend routine screening for RCC in patients who have been on dialysis for longer than 3 years, including subsequent transplant recipients [21]. Some authors have found that screening with computed tomography or ultrasound increases life expectancy by about 1.6 years among 20-year-old patients with a life expectancy of 25 years [22].

## Extra-renal complications in patients undergoing long-term haemodialysis

### Musculoskeletal system

Patients with chronic renal disease and on haemodialysis may develop many different musculoskeletal abnormalities, including secondary hyperparathyroidism, osteomalacia, osteosclerosis, osteoporosis, amyloidosis and a variety of crystal deposition diseases [23].

Other abnormalities related to the biochemical disturbance of chronic renal failure include soft tissue/vascular calcifications and crystalline arthropathies.

Musculoskeletal sequelae predominantly related to dialysis include an aluminium toxicity manifesting as osteomalacia from ingestion of aluminium salts in phosphate-binding antiacids used to control hyperphosphataemia, amyloidosis and destructive spondyloarthropathy.

Dialysis-related amyloidosis is a frequent and major complication in long-term dialysis patients, and it presents with carpal tunnel syndrome, cystic bone lesions, destructive spondylarthropathy, arthritis, periarthritis and systemic organ involvement [24].

**Fig. 7:**
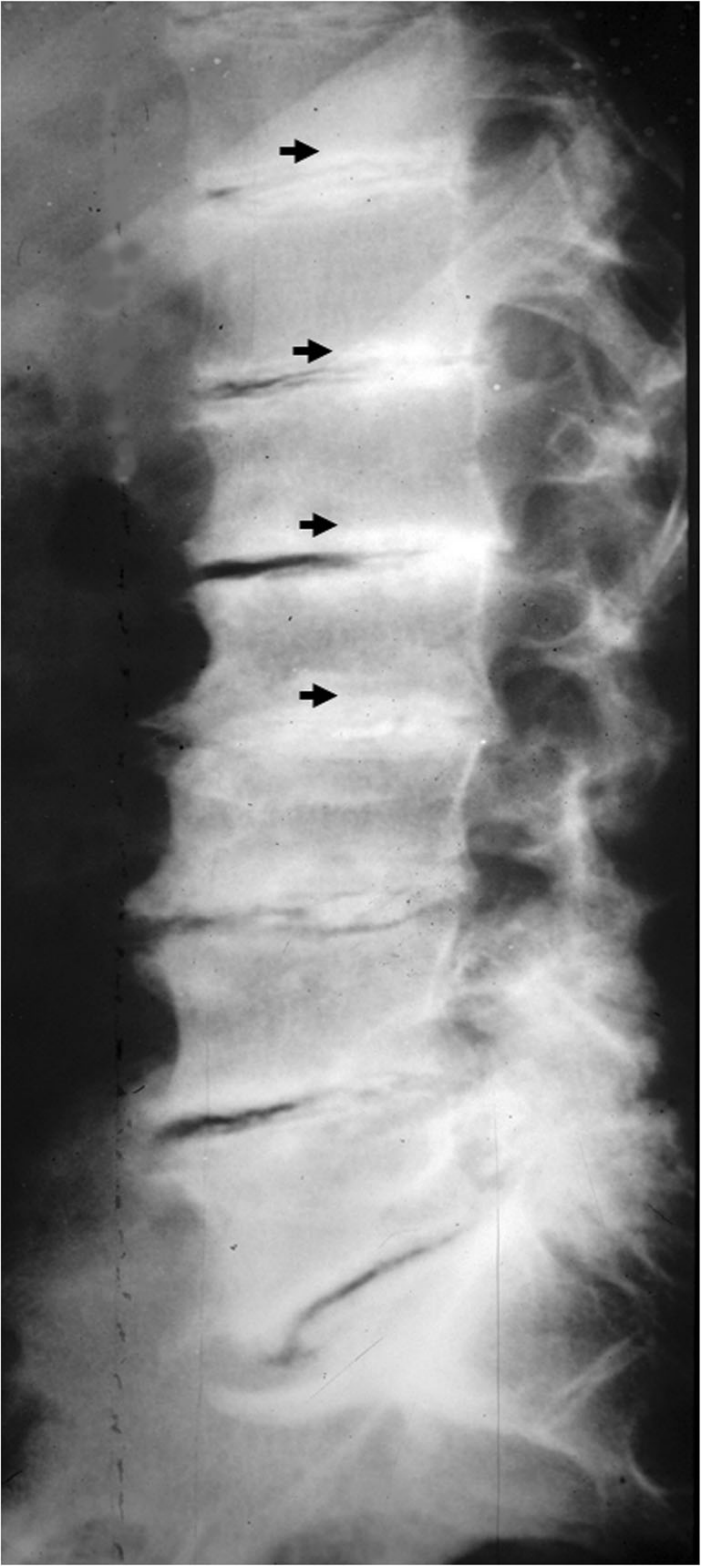
Plain x-ray film of the spine. Diffuse osteosclerosis of the spine in a patient undergoing chronic haemodialysis. Diffuse sub-endplate densities at multiple contiguous levels (*arrows*), a pattern known as “rugger-jersey spine”

Destructive spondyloarthropathy is characterised by rapidly progressive radiographic abnormalities, including the loss of intervertebral disc space, erosion of the subchondral bone in the adjacent vertebral bodies and new bone formation (Fig. 7). In some patients, destructive changes may be severe, simulating infective spondylitis: the persistence of low signal intensity on T2-weighted images generally permits excluding infections [23].

**Fig. 8:**
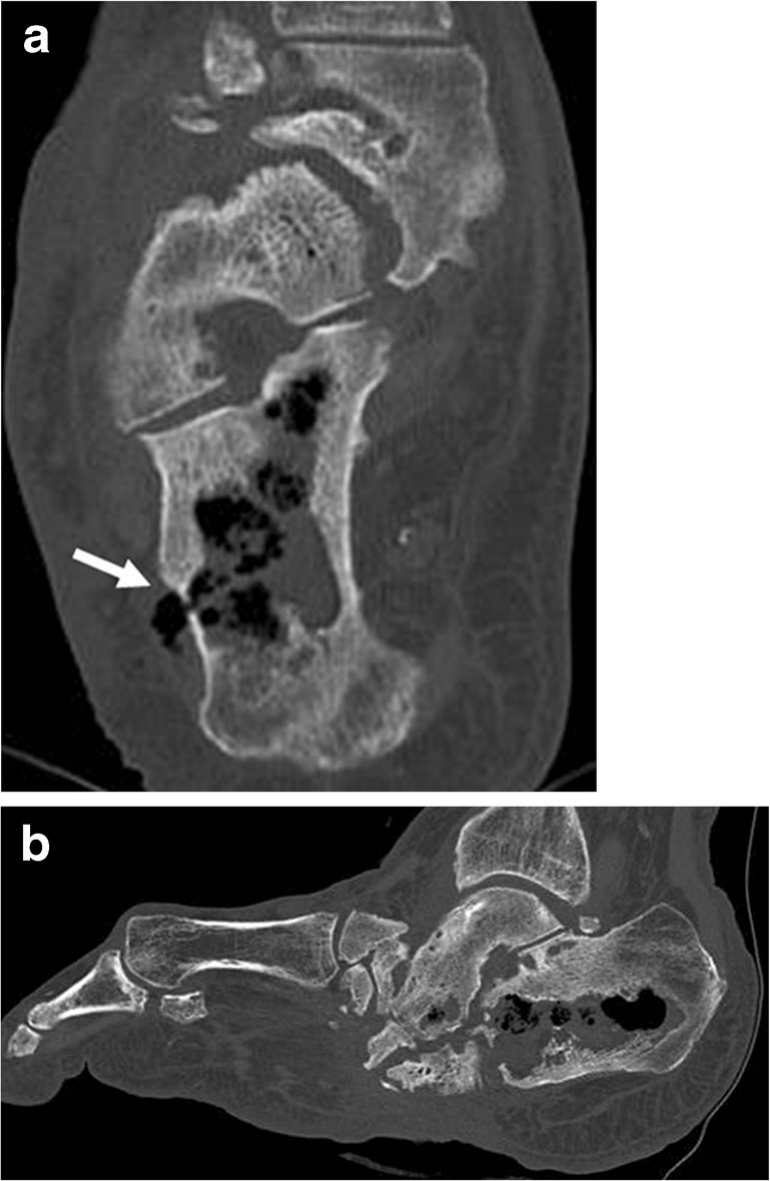
**a**, **b**: Unhenanced CT axial (**a**), sagittal plane. Osteomyelitis of the tarsus in a 72-year-old male with chronic renal failure under dialysis. Extensive bone alterations involving all the tarsal bones with important areas of ostelysis associated with areas of osteosclerosis. The infection extensively involves the calcaneus with intraosseous fluid and gas components. A fistula with subcutaneous soft tissue is visible on the lateral side of the calcaneus (*arrow*)

Avascular necrosis, osteomyelitis (Fig. 8a, b), septic arthritis, tendinosis/tendon rupture and bursitis/synovitis are caused by a combination of chronic renal failure, steroid/immunosuppressants and dialysis [25].

### Renal osteodystrophy

Abnormalities involving the musculoskeletal system are numerous and frequent in patients with chronic renal insufficiency. Chronic renal insufficiency, haemodialysis, peritoneal dialysis, renal transplantation and administration of different medications provoke complex biochemical disturbances of the calcium-phosphate metabolism with a wide spectrum of bone and soft tissue abnormalities named renal osteodystrophy [26]. Renal osteodystrophy is a global term applied to all pathological features of bone in patients with chronic renal failure and it includes osteomalacia or rickets according to the age of the patient, secondary hyperparathyroidism with bone resorption, periosteal reaction, brown tumours, osteosclerosis, osteoporosis, soft tissue and vascular calcifications.

Multiple factors play a role in creating renal osteodystrophy including a deficiency in the active form of vitamin D due to impaired renal metabolism and an increase in the parathormone secretion due to hyperphosphataemia causing hypocalcaemia by precipitating calcium [5].

Radiography still represents the most important and widely performed technique [27] to identify the alterations of renal osteodystrophy. Radionuclide imaging, computed tomography and magnetic resonance imaging are less frequently indicated and are mainly used for more sensitive and precise identifications (aseptic bone necrosis, infection, Looser’s zones), morphological presentations (spinal infection with neural compromise) and the differential diagnosis of complications (destructive spondyloarthropathy versus spinal infection).

**Fig. 9:**
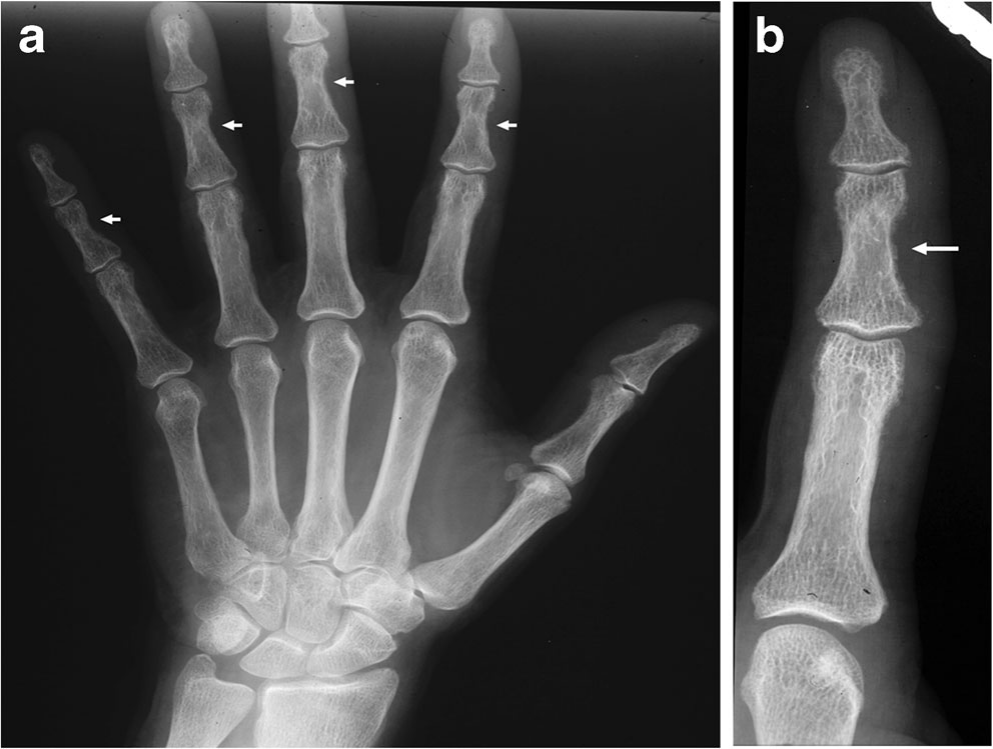
**a**, **b**: **a** Radiographic image of the left hand in a patient undergoing chronic haemodialysis. **b** Magnification. Secondary hyperparathyroidism with bone resorption along the radial aspects of the middle phalanges of the fingers (*arrows*)

The osteoclastic bone resorption affects the surfaces of the bone and may be subperiosteal, intracortical, endosteal, trabecular, subchondral, subligamentous and subtendinous. Early radiographic changes of subperiosteal bone resorption may be demonstrated at the phalangeal tufts and the radial aspects of the middle phalanges of the second and the third hand fingers (Fig. 9a, b) [26]. Early subperiosteal resorption produces the loss of definition of the cortical bone with a characteristic lace-like appearance, which later on progresses to complete cortical disappearance. The advanced bone resorption of the terminal phalanges may result in acro-osteolysis with separation of the tuft and the base of the phalanx. Subperiosteal resorption is frequently combined with intracortical tunnelling and endosteal resorption, which causes scalloped defects of the inner cortical contour. In advanced disease, the widespread trabecular resorption within the medullary bone may be seen with a loss of definition and the disappearance of trabeculae. The most typical manifestation of trabecular bone resorption appears at the cranial vault and is named the ‘salt and pepper’ skull [26].

Subchondral bone resorption may affect different articulations, most commonly the sacroiliac, sternoclavicular and acromioclavicular joints, intervertebral discs and the symphysis pubis. Radiographic changes at the sacroiliac joints are similar to those seen in ankylosing spondylitis and include the widening of the joint space and subchondral bone erosions surrounded by osteosclerosis [26].

Subligamentous and subtendinous bone resorption occurs at numerous sites, especially at the ischial tuberosities, femoral trochanters and the insertions of the coracoclavicular ligaments.

Periosteal new bone production may be seen in patients with advanced hyperparathyroidism affecting the metatarsals, femur, pelvis, humerus, radius, ulna, metacarpals and phalanges.

**Fig. 10:**
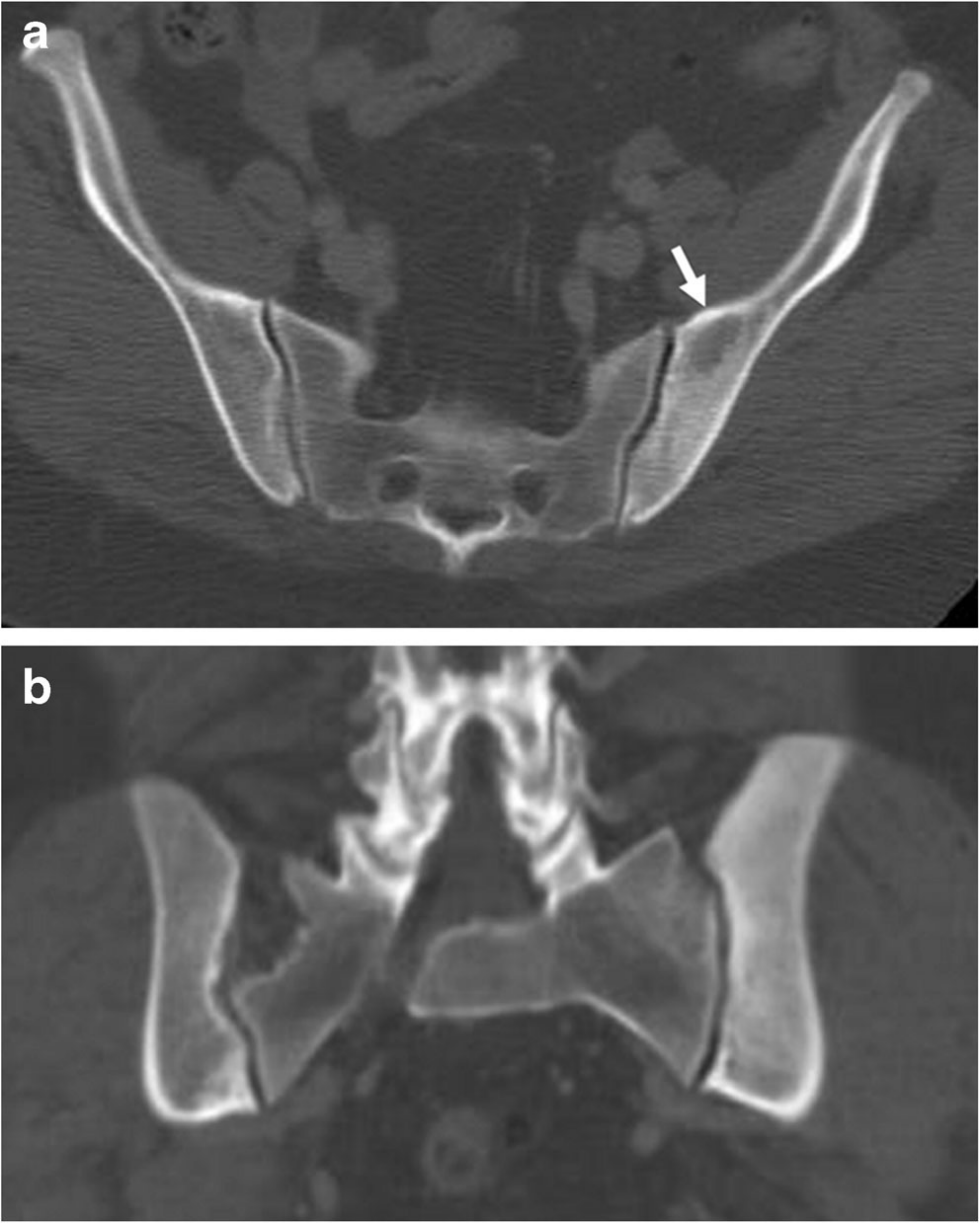
**a**, **b**: Unenhanced CT. Sacral region, axial plane (**a**) and coronal reformation (**b**). Renal osteodystrophy of the left iliac wing with diffuse bone sclerosis and some areas of resorption (*arrows*) due to secondary hyperparathyroidism in a patient undergoing chronic haemodialysis

Osteosclerosis is frequently found in patients with renal osteodystrophy and secondary hyperparathyroidism (Fig. 10a, b). Bone sclerosis in renal osteodystrophy may affect different skeletal elements but it usually predominates in the axial skeleton. One of the typical findings includes broad osteosclerosis localised below the endplates of the vertebral bodies with normal density of the middle parts (‘rugger jersey’ appearance) [26].

Radiographic signs of osteomalacia are a bone density decrease with a loss of definition of the cortical bone and coarsening of the trabecular pattern. Looser’s zones (i.e. Milkman’s fractures, pseudofractures) are rare but pathognomic features, which are most common in the medial part of the femoral neck, pubic rami, ilii, scapulae, ribs and acromion. Looser’s zones represent unmineralised osteoid and are seen as lucent lines perpendicular to the cortex, frequently symmetric in location.

Rickets-like changes may occur in the immature skeleton and reflect an abnormal endochondral ossification. Radiographic features include osteopenia with softening and bending of bones, widening of the growth plate as well as the cupping, expansion, demineralisation and disorganisation of the metaphysis [26].

The osteoporotic bone is characterised by the thinning of cortices and trabeculae. These features are most evident in skeletal elements rich in cancellous bone (spine, proximal femur and forearm). In advanced osteoporosis the loss of trabeculae with pathological fractures may occur. In the vertebral column the disappearance of transverse trabeculae with preservation of the vertical compression trabeculae results in characteristic vertical striations. The final results are pathological fractures with wedge-shaped, biconcave (fish vertebrae) or flattened vertebral bodies and vertebral column deformities. Vertebral fractures can be evaluated subjectively or using quantitative methods. In general, the sensitivity of radiography is poor; 30 % to 50 % of bone tissue has to be lost before osteoporosis can be detected on routine radiographs [26].

### Brown tumours

**Fig. 11:**
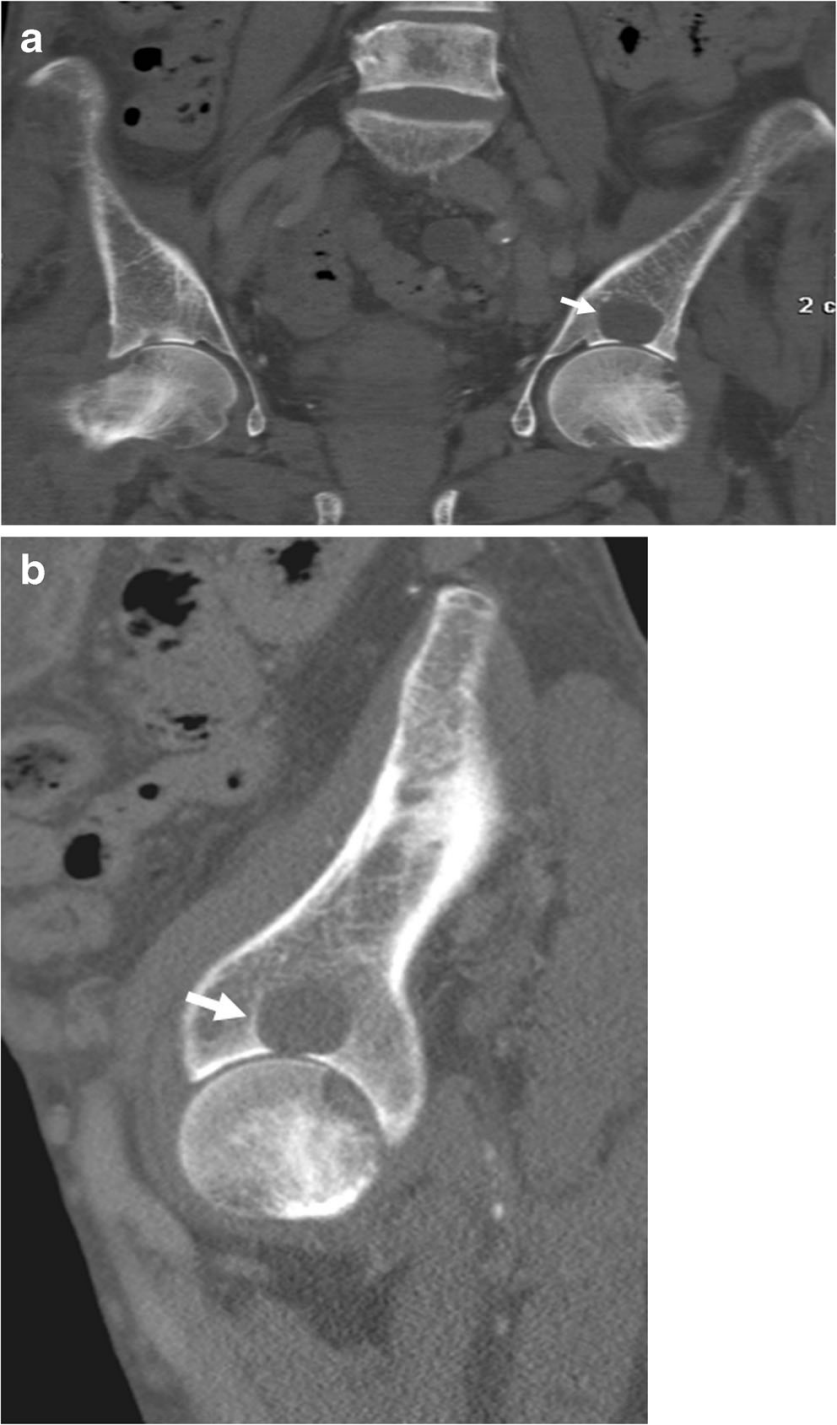
**a**, **b**: Unenhanced CT, coronal (**a**) and sagittal (**b**) reformations. Brown tumour in a patient undergoing chronic haemodialysis with tertiary hyperparathyroidism. The brown tumour appears as a lytic lesion of the ilium (*arrow*)

Brown tumours are a form of cystic fibrous osteitis, the end stage of bone remodelling in primary or secondary hyperparathyroidism [28]. In patients with chronic renal failure brown tumours represent an extreme and serious complication of renal osteodystrophy [28–31]. Brown tumours are caused by the localised replacement of bone by vascularised fibrous tissue (osteitis fibrosa cystica) resulting from parathyroid hormone-stimulated osteoclastic activity [5]. Patients with renal osteodystrophy who have brown tumours actually have autonomously functioning parathyroid glands or tertiary hyperparathyroidism [5]. The fibrous tissue contains giant cells, and the lesions may become cystic, following necrosis and liquefaction [5]. These lesions represent a reparative cellular process rather than a neoplastic process and are located in areas of intense bone resorption (Fig. 11a, b) [5, 32].

Brown tumours occur as single or multiple radiolucent lesions and can be found both within bone and in the adjacent soft tissues. When left untreated, they can undergo central necrosis and cyst formation [5]. Radiographically, brown tumours are well-defined lytic lesions, often eccentric or cortical, that may cause endosteal scalloping and bone expansion.

### Soft tissue and vascular metastatic calcification

Biochemical disturbance of chronic renal failure includes vascular and soft tissue metastatic calcifications and crystalline arthropathies. Vascular calcifications manifest as both medial and intimal calcifications of the arteries and are a hallmark of the accelerated atherosclerosis observed in uraemia [33].

Calcific uraemic arteriolopathy is a rare but life-threatening complication estimated to occur in 1 % of patients with chronic renal disease and in 4.1 % of patients undergoing maintenance haemodialysis [34]. Articular calcinosis is characterised by the deposition of a large amount of calcium around the periarticular regions, and it is commonly noticed over the shoulder, elbow and hip joints [35]. Even visceral (myocardial and pericardial) calcifications may occur in patients undergoing haemodialysis.

**Fig. 12:**
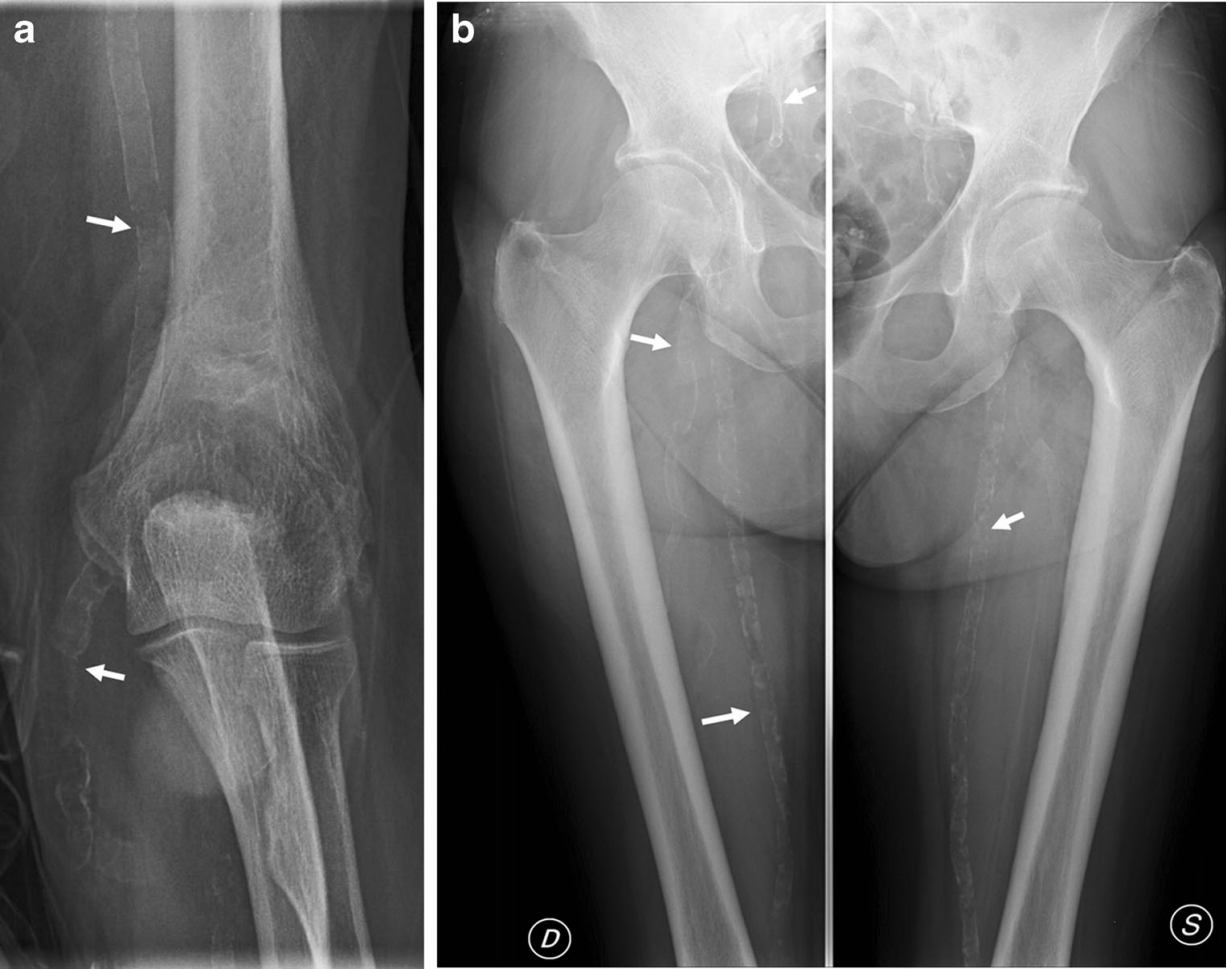
**a**, **b**: Radiographic images of several skeletal segments of different patients (all undergoing long-term haemodialysis) show diffuse calcifications of the peripheral arteries (*arrows*). Vascular calcifications appear as both medial and intimal calcifications of arteries

Vascular calcifications are very common in end-stage renal disease, especially in patients undergoing haemodialysis (Fig. 12a, b) [36, 37]. The mechanisms of vascular calcification are hyperphosphataemia and elevated calcium (Ca) and phosphate (P) products [38, 39].

Vascular calcification induces the stiffening of the vessel wall and reduces the vascular compliance [40]. Iwasa et al. showed a much higher rate of intracranial artery calcification in haemodialysis patients than in the general population, and this finding has been reported to be an independent risk factor for ischaemic stroke. The most frequently involved sites of calcification in haemodialysis patients were the internal carotid artery and the vertebral arteries [41].

### Cardiovascular complications

Cardiovascular complications are common in patients with chronic renal failure and are related to atherosclerosis and hyperlipidaemia, hypertension due to sodium retention and alterations in the renin-angiotensin axis, myocardial dysfunction and pericarditis. More than 50 % of end-stage renal disease patients treated with chronic haemodialysis die from cardiovascular diseases, including congestive heart failure due to the increase of the left ventricular afterload: volume overload between dialyses, hypertension, increased arterial stiffness, anaemia, vascular access flow (arteriovenous fistula) and sympathetic activation. Patients undergoing dialysis present a higher risk of atherosclerosis, also for underlying diabetes mellitus and/or hypertension as causes of end-stage renal disease [4]. Atherosclerosis involves the main and all the peripheral arteries including the intrarenal arteries. Calcifications of the myocardium, coronary arteries and cardiac valves are frequently observed in patients with end-stage renal disease [40, 42].

Cardiovascular calcification lesions can lead to the development of a number of clinically significant complications, including myocardial ischaemia, myocardial infarction, impaired myocardial function, congestive heart failure, cardiac valve insufficiency and cardiac arrhythmias [43, 44]. It has been demonstrated that B2-microglobulin amyloid deposits are extensively localised in the hearts of long-term haemodyalisis patients [45], in particular at the level of the endocardium, myocardium and wall of the small vessels [24].

### Thoracic complications

Patients with chronic renal failure may present several abnormal findings visible on chest radiography due to changes in the phosphorus and calcium metabolism, changes in haemostasis, arterial hypertension, fluid retention or due to dialysis.

The thoracic complications of haemodialysis may be classified into two main topics: cardiovascular and pulmonary [46].

Cardiovascular findings include aortic calcification, cardiomegaly, calcifications of the coronary arteries, pericardial effusion, pulmonary thromboembolism and intra-atrial thrombus [46].

The most frequent pulmonary abnormalities include interstitial and alveolar oedema due to congestive heart failure, pleural effusion, lung atelectasis, metastatic pulmonary calcifications and calcifications of the bronchial walls, pleura and chest wall vessels. Lung parenchymal abnormalities visible on CT include areas of ground-glass opacity, interlobular septal thickening, parenchymal fibrosis scars, emphysema bullae, peribronchovascular interstitial thickening, increased vascular calibre, bronchiectasis, and thickening of the pleura and fissures [46].

The frequency of pulmonary infections in uraemic patients is higher compared to the general population, with evidence of parenchymal consolidation and/or pleural effusion [46].

*Staphylococcus aureus* continues to be the most common (20.8 %) cause of bacterial infection among patients on long-term haemodialysis [46]. Patients on haemodialysis are at increased risk of developing active tuberculosis after primary infection, activation of quiescent disease or reactivation of pre-existing tuberculosis infection [46].

### Neurologic complications

Patients with end-stage renal disease frequently have central nervous system abnormalities, some of them related to end-stage renal disease itself and others related to problems secondary to haemodialysis [47].

The most common neurologic complications in this patient group include focal white matter lesions, cerebral atrophy, dialysis encephalopathy, hypertensive encephalopathy, cerebral infarction, intracerebral haemorrhage, posterior reversible encephalopathy syndrome, osmotic demyelination syndrome, cerebral infection, sinus vein thrombosis and dialysis disequilibrium syndrome. Peripheral neuropathy is also common in patients with chronic renal failure [5].

Acute cerebrovascular disease is one of the most frequent causes of morbidity and mortality in patients on long-term haemodialysis treatment. Cerebral ischaemia and infarction are important risk factors for stroke and are caused by the occlusion of the small, deeply penetrating cerebral arteries. Haemodialysis patients are at greater risk of cerebral haemorrhage because of anticoagulant therapies (e.g., heparin and low-molecular-weight heparins), defects of platelet adhesion, anaemia and inadequate control of hypertension. Cerebral haemorrhage may be intraparenchymal, subdural, epidural or subarachnoid. Patients with end-stage renal disease also have predisposing factors that lead to sinus vein thrombosis.

### Haematologic abnormalities

Haematologic abnormalities are among the most consistent manifestations of uraemia [4]. These abnormalities include anaemia, bleeding and granulocyte and platelet dysfunction. The primary causes of anaemia in chronic renal failure are deficiency of erythropoietin, which is a glycoprotein normally produced by the kidney, and iron deficiency due to a reduced iron intake and frequent blood sampling. Erythropoietin therapy has improved the general status of patients with chronic renal failure. In patients with uraemic syndrome the fat bone marrow is progressively replaced by haematopoietic marrow content with a hypointense appearance on T1-weighted MR images in various anatomical regions (especially the spine and upper and lower limb bones). A haemorrhagic diathesis is common in patients with chronic renal failure. Spontaneous non-traumatic bleeding may affect the perinephric and subcapsular spaces, renal parenchyma or collecting system. Gastrointenstinal bleeding is also common in uraemic patients [5].

### Encapsulating peritoneal sclerosis

Although medical considerations may dictate the choice of continuous ambulatory peritoneal dialysis (CAPD) or haemodialysis for some patients, most cases of renal failure are treatable with either method.

Therefore, it is important to recognise some potentially fatal complications of CAPD that must be detected at an early stage in order to allow a prompt interruption of CAPD and direct the patient toward the haemodialysis treatment only.

Encapsulating peritoneal sclerosis is an uncommon serious complication of CAPD [48] associated with significant morbidity and mortality.

In a large study by Rigby and Hawley [49], the overall prevalence of encapsulating peritoneal sclerosis is 0.7 %; however, the prevalence increases to 19.4 % in patients who have been on CAPD [49] for more than 8 years.

Encapsulating peritoneal sclerosis is a condition characterised by fibrotic thickening of the peritoneum, which can progress to the encasement or encapsulation of the small-bowel loops with a resultant bowel obstruction. The peritoneal thickening can be smooth or irregular and nodular, and mild peritoneal thickening typically progresses to diffuse, severe peritoneal thickening and eventually peritoneal calcification.

Peritoneal thickening progresses to peritoneal encapsulation of the involved small-bowel loops, a process that has been described as the “cocooning” of the small bowel, by a sheath of fibrous, sclerosed peritoneum.

The small-bowel loops are often centrally collected by encapsulating the fibrotic peritoneum. The fibrotic process may appear as a mass of small bowel loops tethered together. Ultimately, small-bowel necrosis with perforation may occur.

In encapsulating peritoneal sclerosis, the fibrotic, thickened peritoneal membranes can result in loculated ascites. Since the small bowel becomes involved, tethered and mass-like, the tethered small bowel may also produce loculated fluid collections.

Numerous other complications related to peritoneal dialysis have been described, including catheter dysfunction, haematoma, dialysate leak and hydrothorax [50].

### Dialysis access complications

Repeated access to the circulation is essential to perform an adequate maintenance haemodialysis. End-stage renal failure patients requiring long-term haemodialysis need a durable vascular access, and the maintenance of an acceptable vascular access is an important issue for patients with renal failure. Haemodialysis is usually performed with an internal arteriovenous fistula (AVF) or polytetrafluoroethylene (PTFE, Teflon) graft, also known as a prosthetic bridge graft (PBG). Surgically constructed arteriovenous (AV) fistulas (radio- or brachiocephalic), synthetic AV grafts or a venous catheter positioned in a central vein are common means of establishing a vascular access for long-term haemodialysis.

Vascular stenosis is the major cause of access failure. The treatment of stenosis with percutaneous interventions, angioplasty, endoluminal vascular stents or thombolysis as an alternative to surgery has been shown to increase the chance of survival for patients with AVF and PBG dialysis [51]. Several surveillance techniques can be used for the prompt detection of access stenosis development. Colour Doppler US [52] and digital subtraction angiography [53] are most commonly used for the detection of access stenosis, while contrast-enhanced multi-detector CT [54] and MR angiography of shunts [55] have recently been introduced. Angiographic evaluation via puncture of the brachial artery is indicated when an interventional procedure is planned.

### Arteriovenous fistulas

The AV fistula is considered the best long-term vascular access for haemodialysis because it provides adequate blood flow, is long-lasting and has a lower complication rate than other types of access. A nephrologist or surgeon creates an AV fistula. As a result, the vein grows larger and stronger, making repeated needle insertions for haemodialysis treatments easier. AV fistulas also present some disvantages, including the incomplete maturation of the vein, and a 1–4-month period may be required before the AV fistula can be used. Fistula dysfunction is the most common reason for a second intervention and recurrent hospitalisation. The rate of AVF complications increases with age and the erythropoietin dose as well as among patients with a history of a previous failed shunt [56]. The clinical symptoms include poor flow with loss of thrill in the graft, enlarging pseudoaneurysm or ipsilateral limb swelling.

**Fig. 13:**
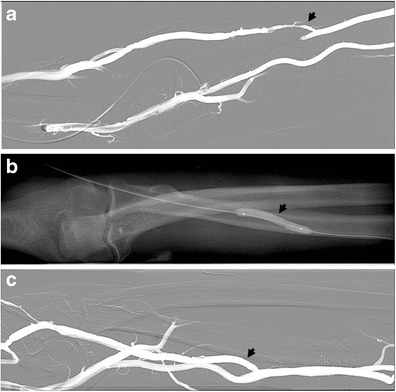
**a**–**c**: An 80-year-old male patient with chronic renal failure undergoing haemodialysis. Stenosis (about 5 cm in length) of outflow of AVF of the arm (*arrow*) (**a**), treated by angioplasty (**b**) with recovery of the normal patency (**c**)

**Fig. 14:**
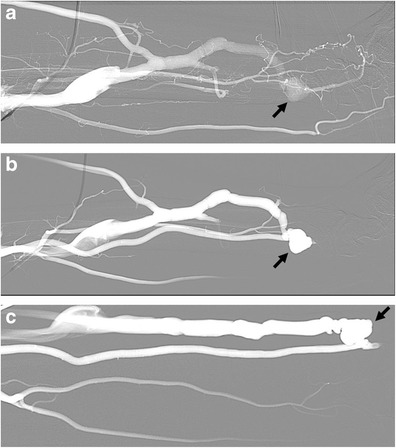
**a**–**c**: AVF of the forearm at the level of the metaphysis of the radius in a 70-year-old patient with chronic renal failure under dialysis. Outflow perianastomotic pseudoaneurysms (*arrows*). Vein aneurysms or pseudoaneurysms are located in the puncture site

The main complications of AV fistula include thrombosis with occlusion or stenosis of the venous outflow (Fig. 13a, c) as well as aneurysm or pseudoaneurysm of the venous tract (Fig. 14a, c) close or distal to the anastomosis with the radial artery [57]. In particular, the venous outflow obstruction is the most common cause of AVF thrombosis and poor dialysis. Most typically, pseudoaneurysms are found in areas of repeated puncture or surgical anastomosis.

Complications of the arterial tract of AVFs are rare. Proximal arterial disease, the occlusion or arterial anastomotic stenosis are readily shown by digital subtraction angiography. The arterial stenosis appears as a narrowed area adjacent to or between areas of graft ectasia. The laceration of the radial artery may determine a haematoma with compression of the nervous structures in the carpal tunnel.

### Arteriovenous grafts

The AV fistula with its long patency rate and low complication profile is usually the first choice for vascular access creation. However, when superficial veins are not suitable for the AV fistula creation, or they have all been exhausted as a result of repeated AV fistula procedures, arteriovenous grafts using expanded polytetrafluoroethylene (PTFE) are an alternative [58]. The PTFE graft—prosthetic bridge graft (PBG)—consists of a synthetic catheter inserted and sutured into an artery, usually the radial artery, and a vein, usually the cephalic or the basilic vein.

Unlike the arteriovenous fistula used for chronic haemodialysis, the AV graft can be used immediately. Just as in natural AVF, the non-dominant arm should be used first [58]. Compared to properly formed fistulas, grafts tend to have more problems with clotting and infections and require earlier replacement. However, a well-cared-for graft can last several years.

The overall complication rate for AV grafts is twice as that of AV fistulas. The most common complication associated with the AV graft is venous outflow obstruction with graft thrombosis, which accounts for 85 to 90 % of problems with haemodialysis. PBGs have a shorter mean patency than native AVFs. The most common causes of failure include the surgical twisting or kinking of the graft during implantation, arterial plugs, stenosis of the venous anastomosis or an unsuspected venous stenosis. Venous stenosis results in problems that have the net effect of causing inadequate dialysis. Stenosis most frequently involves the venous anastomosis, but it may occur anywhere within the system composed by the graft, anastomosis and its draining veins. Complete thrombosis is easily identified when it involves the graft or the entire venous limb. Moreover, AV grafts may produce complications related to arterial inflow stenosis, arterial anastomotic stenosis, intragraft stenosis, graft pseudoaneurysm and graft degeneration. Treatment options are either a surgical revision or endovascular stent graft placement.

## Dialysis access catheters

The high thrombosis rate of AVF and PBGs and the high recurrence rates after thrombolysis and angioplasty have led to consideration of performing haemodialysis with percutaneously inserted venous catheters. Nowadays, venous catheters are often taken into consideration as a long-term solution for haemodialysis because of the improvement in biomaterials available for catheters. Percutaneous venous catheters present the advantage of being placed on an outpatient basis; moreover, they do not require any maturation period, and they prevent repeated punctures of the AVF. The disavantages are represented by the fact that venous catheters are cosmetically less acceptable, and the achievable peak of flow rates are lower than with AVFs and PBGs.

A venous catheter positioned in a central vein (usually the right internal jugular vein or, less frequently, the femoral or subclavian veins) through the subclavian vein for a temporary access has two chambers to allow a two-way flow of blood. Once a catheter has been placed, the needle insertion is not necessary. Catheters are not ideal for permanent access, and their patency is maintained via a heparin-saline infusion by flushing the catheter with 2 or 3 ml of heparin (2,000–3,000 units) twice daily (every 12 h). They can clog, become infected, and cause stenosis or complete thrombosis of the central vein in which they are placed. However, if there is a need to starting haemodialysis immediately, a catheter will work for several weeks or months while the permanent access is gradually developing.
